# Anti-Inflammatory Activities of Ocotillol Isolated from *Tetragonula iridipennis* Propolis: A Study on In Vitro and In Silico Models

**DOI:** 10.3390/ph19030361

**Published:** 2026-02-25

**Authors:** Nguyen Thanh Cong, Nguyen Van Phuong, Do Van Hieu, Nguyen Hoang Viet, Le Nguyen Thanh

**Affiliations:** 1 Department of Pharmacognosy, Faculty of Pharmacognosy and Traditional Medicine, Hanoi University of Pharmacy, Hanoi 11000, Vietnam; congnt@dainam.edu.vn; 2 Faculty of Pharmacy, Dai Nam University, Hanoi 10000, Vietnam; 3 National Institute of Medicinal Materials (NIMM), Hanoi 11018, Vietnam

**Keywords:** *Tetragonula iridipennis*, ocotillol, anti-inflammatory, nitric oxide inhibition, network pharmacology, molecular dynamics

## Abstract

**Background/Objectives**: This study evaluated the anti-inflammatory effects of ocotillol, a compound isolated from the ethanol extract of propolis of the *Tetragonula iridipennis* stingless bee. Through its ability to inhibit NO production in an in vitro model, it investigated the NO inhibition mechanism using network pharmacology combined with molecular docking. **Methods**: The NO production inhibitory activity was determined by colorimetric assay using Griess reagent. An in silico study was performed using network pharmacology analysis, molecular docking, and molecular dynamics simulations. **Results**: The in vitro results demonstrated that ocotillol exhibited significant anti-inflammatory effects by effectively inhibiting NO production, with an IC_50_ value of 20.29 ± 2.1 µg/mL. The network pharmacology analysis revealed that ocotillol targets 14 molecular sites related to NO, with TACR1 showing the best binding affinity at −10.0 kcal/mol. Molecular dynamics simulations suggest that TACR1 is a potential target. As indicated by the stable interaction profile, further validation in complex biological membranes is warranted. **Conclusions**: This study also provides evidence for the correlation between in vitro and in silico models, thus laying the groundwork for in vivo evaluations to confirm the anti-inflammatory mechanism of ocotillol.

## 1. Introduction

Nitric oxide (NO), a gaseous free radical, is a product of the conversion of L-arginine by a group of enzymes known as nitric oxide synthase (NOS) [1]. Since its discovery, NO has become a significant subject of research due to its ability to act as a unique gene signaling molecule in various physiological and pathological processes [2,3,4]. NO not only functions as a vasodilator in the cardiovascular system but is also implicated in other pathological conditions such as infections, fetal development, and postnatal growth, angiogenesis, hypertrophy, and programmed cell death (apoptosis) [5]. Specifically, in the context of inflammation, inducible nitric oxide synthase (iNOS), stimulated by cytokines, produces a large amount of NO [6]. Therefore, NO remains a potential subject for evaluating the anti-inflammatory properties of new sources.

**Figure 1:**
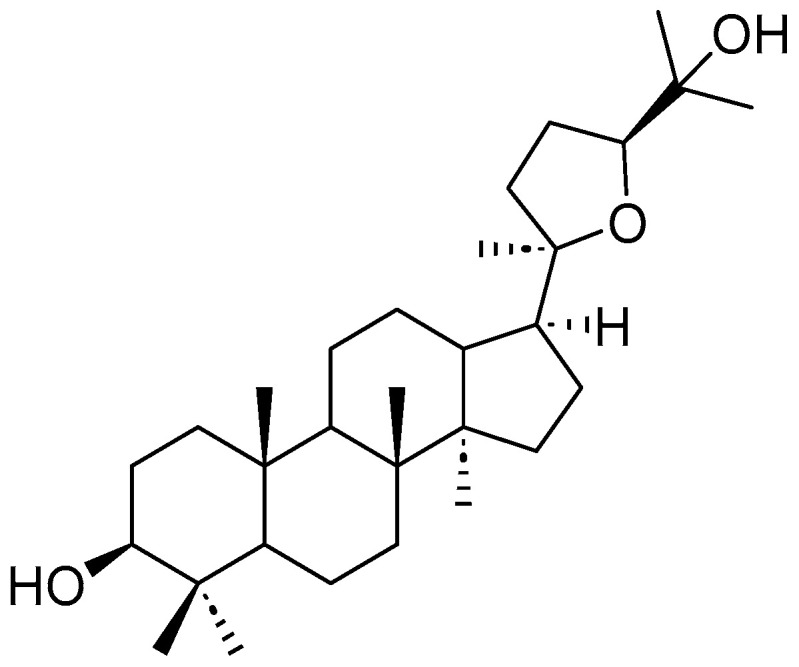
The chemical structure of ocotillol.

*Tetragonula iridipennis* is a stingless bee species that plays a crucial role in maintaining the rich biodiversity of Vietnam’s ecosystem. In a study conducted by Diep Thi Lan Phuong and colleagues, the research team successfully isolated sixteen compounds from the ethanol extract of propolis from *Tetragonula iridipennis*, including ocotillol (Figure 1) [7]. Additionally, a preliminary study conducted by the group indicated that the ethanol extract of this propolis exhibited anti-inflammatory effects by effectively inhibiting NO production.

Ocotillol and its derivatives have been reported to exhibit cardioprotective, enhance neuronal activity, reduce drug resistance in cancer cells, and have antibacterial properties [8,9,10,11]. Furthermore, ocotillol has been reported to exhibit significant anti-inflammatory effects in colitis models and suppress the release of inflammatory mediators, such as IL-6 and TNF-α, thereby mitigating tissue damage in sepsis [12]. Currently, there are no studies evaluating the anti-inflammatory potential of this compound through NO inhibition. Previous studies assessing the NO inhibitory effects have focused on NOS, which is incomplete and inaccurate. Therefore, in this study, we aim to evaluate the NO inhibitory activity in vitro and investigate the NO inhibition mechanism using network pharmacology combined with molecular docking.

## 2. Results and Discussion

### 2.1. NO Inhibition Activity of Ocotillol

The results of the NO inhibition assay showed that ocotillol had an IC_50_ value of 20.29 ± 2.1 µg/mL compared to the positive control N^G^-Methyl-L-arginine acetate (L-NMMA), which had an IC_50_ value of 5.71 ± 0.5 µg/mL (Table 1). To ensure that the observed NO inhibition was not a consequence of cytotoxicity, an MTT assay was performed. Ocotillol maintained cell viability above 90% at concentrations up to 32 µg/mL, supporting the conclusion that its anti-inflammatory effect is pharmacologically specific. These results suggest that ocotillol exhibits promising anti-inflammatory activity through the inhibition of nitric oxide production in an in vitro model. Nitric oxide plays a crucial role as a mediator in inflammatory pathways because elevated NO levels lead to stimulatory responses, resulting in inflammatory effects. Therefore, inhibiting NO synthesis can significantly reduce overall inflammatory responses. The promising anti-inflammatory results of the ocotillol compound through NO production inhibition in an in vitro model indicate the necessity for further in-depth research using network pharmacology combined with molecular docking to identify related molecular targets. This forms the basis for subsequent evaluations in in vivo models for this compound.

### 2.2. Results of Data Preparation

Using the GeneCards (Supplementary S1) and OMIM databases (Supplementary S2), after removing duplicates, a total of 392 genes related to NO biosynthesis were identified.

**Figure 2:**
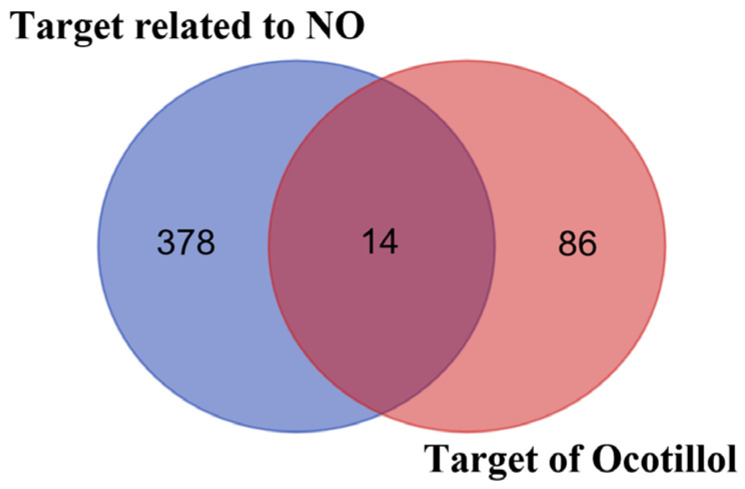
A Venn diagram representing ocotillol.

In the next step, the structures of the ocotillol were converted into SMILES format and subsequently searched for targets in the SwissTarget (Supplementary S3) and STITCH databases. After removing duplicates, 100 potential targets were obtained. The Venn diagram in Figure 2 shows that 14 targets (Supplementary S4) are associated with the “target related to NO” of the “target of ocotillol” (Table 2).

### 2.3. GO Analysis and KEGG Pathway Analysis

**Figure 3:**
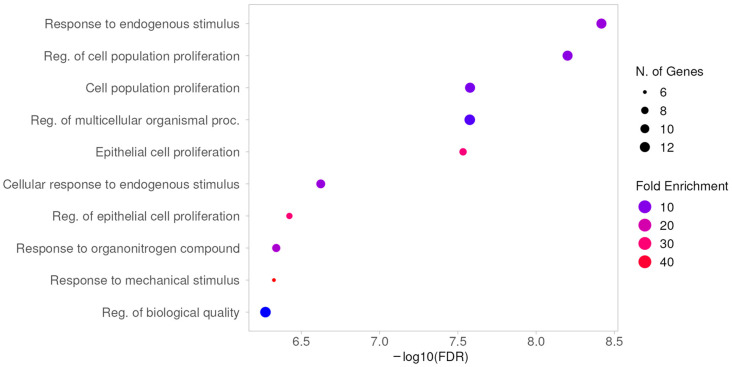
GO analysis: biological processes.

To further clarify the biological functions and potential signaling pathways of the common genes between ocotillol and NO biosynthesis, Gene Ontology (GO) and Kyoto Encyclopedia of Genes and Genomes (KEGG) analyses were performed. The GO enrichment chart displays the top 10 enriched categories in biological processes (BP), cellular components (CC), and molecular functions (MF), in ascending order of FDR values. In the BP analysis, 14 common targets were enriched in biological processes such as response to endogenous stimulus, regulation of cell population proliferation, cell population proliferation, regulation of multicellular organismal processes, epithelial cell proliferation, cellular response to endogenous stimulus, regulation of epithelial cell proliferation, response to organonitrogen compound, response to mechanical stimulus, and regulation of biological quality (Figure 3).

**Figure 4:**
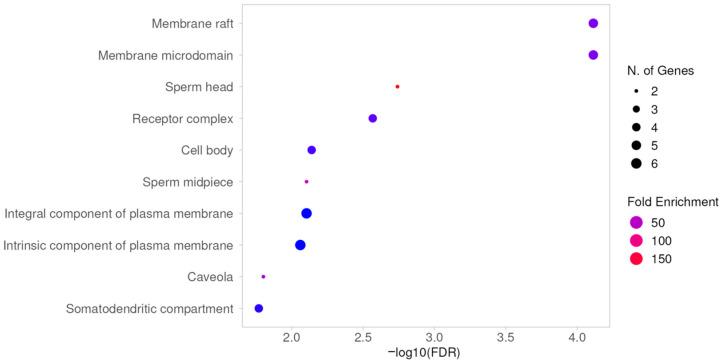
GO analysis: cellular components.

The CC analysis showed these common targets were involved in the membrane raft, membrane microdomain, sperm head, receptor complex, cell body, sperm midpiece, integral component of plasma membrane, intrinsic component of plasma membrane, caveola, and somatodendritic compartment (Figure 4).

**Figure 5:**
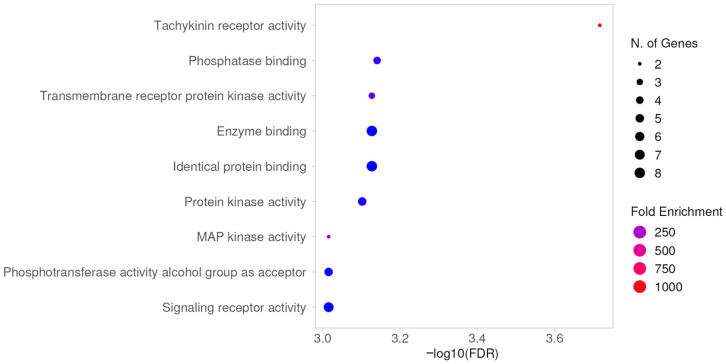
GO analysis: molecular function.

Meanwhile, the MF analysis indicated that the molecular functions of these 14 common targets were mainly related to tachykinin receptor activity, phosphatase binding, transmembrane receptor protein kinase activity, enzyme binding, identical protein binding, protein kinase activity, MAP kinase activity, phosphotransferase activity with alcohol group as acceptor, and signaling receptor activity (Figure 5).

**Figure 6:**
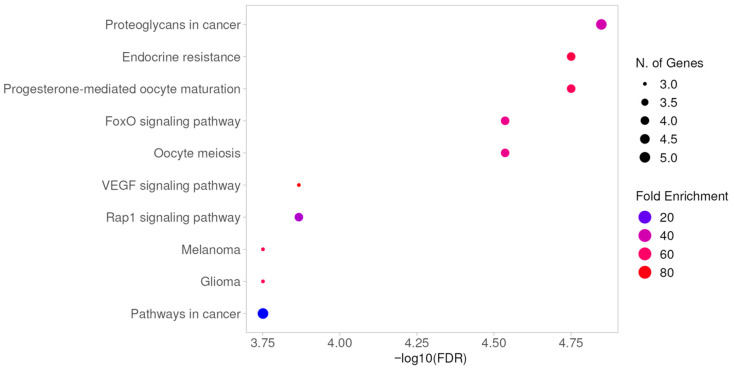
KEGG pathway analysis.

Furthermore, the common signaling pathways of the compound were analyzed through KEGG. The top ten significant pathways were selected with a threshold of *p* < 0.05 (Figure 6). These common targets impact pathways related to proteoglycans in cancer, endocrine resistance, progesterone-mediated oocyte maturation, FoxO signaling pathway, oocyte meiosis, VEGF signaling pathway, Rap1 signaling pathway, melanoma, glioma, and pathways in cancer. Proteoglycans are involved in regulating the cellular microenvironment and can promote inflammation, thereby facilitating cancer development [13,14]. Meanwhile, the VEGF signaling pathway plays a crucial role in neovascularization, where chronic inflammation can activate this pathway, leading to increased angiogenesis in cancerous tumors [15,16]. The Rap1 signaling pathway can influence cell adhesion integrity and inflammation, potentially leading to inflammation and cancer progression [17,18]. These three pathways all impact the development and metastasis of malignant tumors and gliomas, which are extensively studied types of cancer with many associated signaling pathways. The FoxO signaling pathway regulates apoptosis, cell cycle progression, and insulin resistance, all of which affect inflammation and cancer [19,20].

### 2.4. Molecular Docking Studies

**Figure 7:**
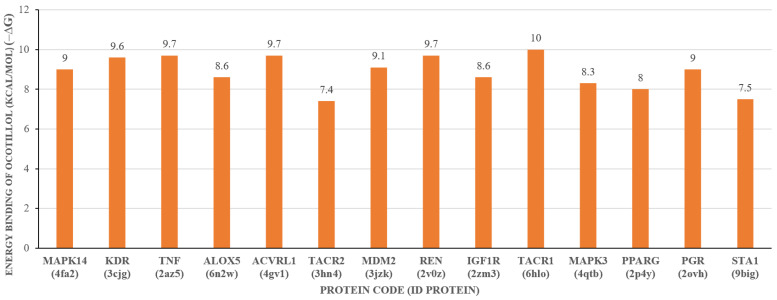
Docking results of ocotillol with 14 protein targets.

**Figure 8:**
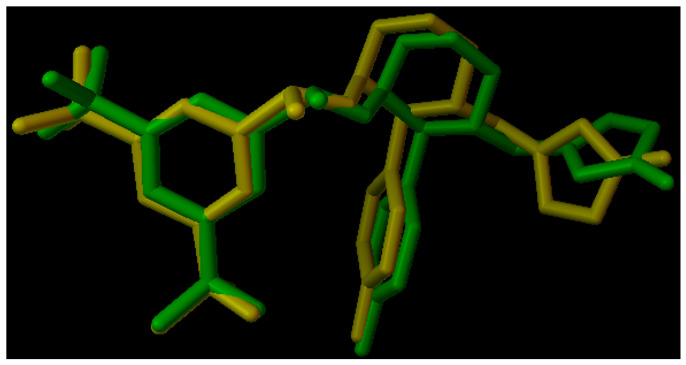
RMSD value. The co-ligand is presented in yellow, and the redocked co-ligand is shown in green.

The results indicate that ocotillol interacts with 14 molecular targets; however, it is not yet possible to determine whether the interactions are strong or weak. Therefore, the next step will involve using molecular docking methods to study the binding affinity and binding mode. In this study, we utilized molecular docking techniques to identify targets with the lowest binding energy to ocotillol, aiming to screen and select molecular targets for further research. AutoDock Vina software version 1.5.7 was used to determine the binding energy between the compound and 14 common protein targets. The binding energy of the compound and 14 protein targets are presented in Figure 7, with parameters including box dimensions of 40 Å in OX, OY, OZ, an energy range of 20, and an exhaustiveness of 16. Moreover, the RMSD value of the co-ligand during the docking process is measured at 1.096 Å, as illustrated in Figure 8, underscoring the precision and reliability of the employed research methodology.

The docking results in Figure 7 showed that the ocotillol has strong binding affinity with the Neurokinin 1 receptor (TACR1; ID: 6HLO), TNF-*α* (TNF; ID: 2AZ5), Renin (REN; ID: 2V0Z), and Vascular endothelial growth factor receptor 2 (KDR; ID: 3CJG), with binding energies of −10.0 kcal/mol, −9.7 kcal/mol, −9.7 kcal/mol, and −9.6 kcal/mol, respectively.

**Figure 9:**
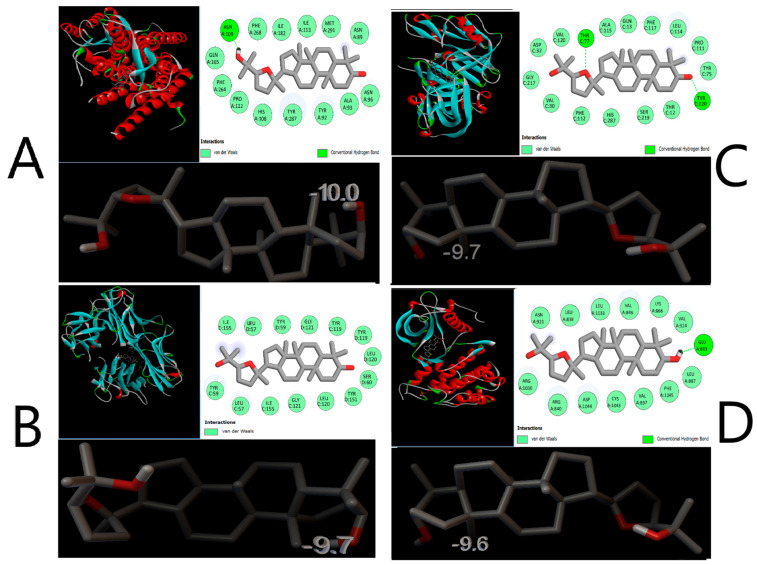
The binding energy and binding modes of ocotillol with proteins. TACR1 (**A**), TNF (**B**), REN (**C**), and KDR (**D**). In 3D models, alpha helices are shown in red, beta sheets in teal, and loops in green. In 2D diagrams, residues involved in van der Waals interactions are represented by light green circles, while conventional hydrogen bonds are indicated by lime green circles and dashed lines. Interaction energies (kcal/mol) are displayed in the 3D views for clarity.

The binding mode analysis indicates that the ocotillol forms hydrogen bonds with the amino acid ASN A:109 and Van der Waals interactions with ile182, gln165, ile113, phe264, asn89, met291, ala93, his108, tyr92, tyr287, asn96, pro112, and phe268 with the TACR1 protein (Figure 9A).

The compound also forms Van der Waals interactions with the amino acids ile155, leu57, tyr59, gly121, tyr119, tyr119, leu120, ser60, tyr151, tyr59, leu57, ile155, gly121, and leu120 of the TNF-*α* protein (Figure 9B).

For the renin protein (Figure 9C), ocotillol forms Van der Waals interactions with asp32, val120, ala115, gln13, phe117, leu114, pro111, tyr75, gly217, val30, phe112, his287, ser219, and thr12, and hydrogen bonds with two amino acids, thr77 and tyr220.

Finally, ocotillol forms Van der Waals interactions with the amino acids asn921, leu838, leu1033, val846, lys866, val914, leu887, phe1045, val897, cys1043, asp1044, arg840, and arg1030, and a hydrogen bond with glu883 of the vascular endothelial growth factor receptor 2 protein (Figure 9D).

The protein target TACR1 is closely associated with nitric oxide (NO) in inflammatory processes [21,22]. Studies have demonstrated that the use of antagonists to inhibit TACR1 results in reduced NO levels, evidenced by decreased concentrations of 3-nitrotyrosine, a marker of NO production [23,24]. Furthermore, TACR1 is involved in the pathogenesis of inflammatory diseases such as inflammatory bowel disease and irritable bowel syndrome by regulating the differentiation and function of CD4 T cells [25]. Additionally, in systemic inflammation, TACR1 enhances the expression of cyclooxygenase-2 protein in the early phase [26], while NO acts as a versatile mediator in inflammatory pathology. Combined with the binding energy results presented in Figure 8, this study selects TACR1 as a target for further evaluation of its anti-inflammatory potential based on NO inhibition.

### 2.5. Molecular Dynamics Studies

To thoroughly investigate and analyze the stability of the interaction between the ocotillol and the protein (TACR1), molecular dynamics (MD) simulations were conducted and evaluated based on Root Mean Square Deviation (RMSD) (Supplementary S5), Root Mean Square Fluctuation (RMSF) (Supplementary S6), Solvent Accessible Surface Area (SASA) (Supplementary S7), and Radius of Gyration (RG) (Supplementary S8).

**Figure 10:**
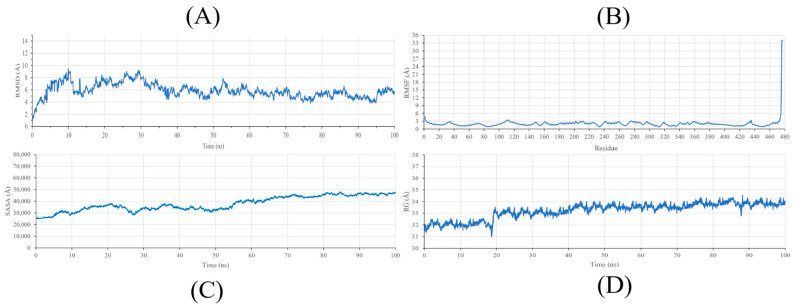
RMSD (**A**), RMSF (**B**), SASA (**C**), RG (**D**) of protein TACR1.

Root Mean Square Deviation (RMSD) is crucial in providing comprehensive insights into the structural changes in the complex during interaction. The RMSD plot (Figure 10A) indicates that the RMSD values of the protein exhibit significant fluctuations within the first 30 ns, ranging from 4 to 9 Å, which can be attributed to the structural adjustment of the receptor upon ligand binding in an aqueous environment. However, in the subsequent 70 ns, these values stabilize within the range of 4 to 6 Å, suggesting that the protein-ligand complex reaches a state of relative equilibrium for the majority of the simulation period.

Furthermore, the RMSF plot (Figure 10B) displays consistent flexibility patterns among individual amino acids during the interaction process. Notably, most amino acids exhibit fluctuations below 3 Å, with the majority of amino acids interacting with the Ocotillol at positions 64 (tyr92), 65 (ala93), 85 (ile113), 137 (gln165), and 154 (ILE182) remaining below 2 Å, indicating a high level of stability throughout the simulation. Although a significant peak of approximately 35 Å was observed at the C-terminus, this is interpreted as the inherent flexibility of the terminal loops in a solvent-exposed environment rather than a disruption of the protein’s core structure. These results indicate that despite the flexibility of peripheral regions, the functional domains relevant to ocotillol binding remain structurally robust.

SASA (Solvent Accessible Surface Area) is a crucial parameter in studying molecular interactions with solvents. The results of investigating the solvent-accessible surface area of a molecule help to better understand the degree of interaction between the molecule and its surrounding environment. Figure 10C shows the SASA values for the TACR1-ocotillol complex ranged between 25,000 and 50,000 Å^2^ throughout the simulation. The maintenance of a stable SASA profile suggests that the protein does not undergo significant unfolding or drastic conformational shifts during the interaction. This consistency supports the structural integrity of TACR1, facilitating the formation of persistent non-bonded interactions, such as van der Waals forces, between ocotillol and the receptor’s hydrophobic regions.

Additionally, the Radius of Gyration (Rg) was analyzed to determine the structural compactness of the protein during the simulation (Figure 10D). The Rg values remained relatively constant, fluctuating slightly between 31 and 34.5 Å. Unlike general flexibility markers, the steady Rg profile indicates that TACR1 maintains a compact folded state while bound to Ocotillol. This sustained compactness, in consistency with the SASA and RMSD data, suggests that the protein-ligand complex is energetically favorable and structurally integrated. These parameters collectively support the hypothesis that Ocotillol effectively stabilizes the target protein, providing a reliable basis for its predicted anti-inflammatory mechanism.

## 3. Materials and Methods

### 3.1. Materials

Ocotillol was isolated from the propolis of *Tetragonula iridipennis*, as previously described in our group’s chemical constituent study [7].

### 3.2. NO Inhibition Assay

RAW 264.7 (ATCC^®^ -TIB-71TM) cell line was cultured in DMEM (Gibco, Life Technologies Corporation, Grand Island, NY, USA) with supplements at 37 °C and 5% CO_2_. Cells were placed in a 96-well plate at a density of 2 × 10^5^ cells per well and incubated for 24 h. After incubation, the medium was replaced with FBS-free DMEM for 3 h. The cells were then treated with varying concentrations of the test sample for 2 h before LPS stimulation at 10 μg/mL for 24 h to induce nitric oxide production. Nitrite (NO_2_^−^) levels, indicative of NO generation, were quantified using Griess reagents (ThermoFisher, Waltham, MA, USA) by mixing 100 µL of the culture medium with 100 µL of Griess reagent in a new 96-well dish and incubating for 10 min, after which nitrite concentration was measured spectrophotometrically at 540 nm. Control wells contained only culture medium, while LPS-treated wells served as positive controls. N^G^-methyl-L-arginine acetate (L-NMMA. Sigma-Aldrich, St. Louis, MO, USA) was used as a positive control in this study. Nitrite levels for each sample were calculated using a standard curve from sodium nitrite (NaNO_2_). The inhibitory effect of the sample on NO production was evaluated using the following formula, with OD_(+)_ being the optical density of the positive control and OD_(−)_ being the optical density of the negative control [27,28,29,30].

Percentage of NO inhibition (%) = (OD_(+)_ − OD_sample_)/(OD_(+)_ − OD_(−)_) × 100%

### 3.3. Cell Viability Assay (MTT Assay)

To ensure that the observed nitric oxide inhibition was not a result of non-specific cytotoxicity, cell viability was evaluated using the 3-(4,5-dimethylthiazol-2-yl)-2,5-diphenyltetrazolium bromide (MTT) colorimetric assay. After the initial incubation period, 10 µL of MTT reagent (5 mg/mL) was added to each well, and the plates were incubated for an additional 4 h at 37 °C. Subsequently, the culture medium was carefully removed, and the resulting formazan crystals were dissolved in 100 µL of dimethyl sulfoxide (DMSO, Merck, Damstadt, Germany). The optical density (OD) was measured using a microplate spectrophotometer at a wavelength of 540 nm. Wells containing only culture medium served as the negative control, while wells containing untreated cells (representing 100% growth) served as the positive control. The percentage of cell viability was calculated according to the following formula [27,28,29,30]:

Cell Viability (%) = 100% − [(OD_(+)_ − OD _sample_)/(OD_(+)_ − OD_(−)_) × 100%]

### 3.4. Data Preparation

The genes related to NO biosynthesis were collected from the GeneCards database, accessible at the following URL: https://www.genecards.org (accessed on 7 March 2024) [31], and the OMIM database, available at https://www.omim.org (accessed on 7 March 2024) [32], using the searching keyword “Nitric oxide synthesis”. In the next phase, the compound Ocotillol was screened for its target effects using the SwissTargetPrediction web server (http://www.swisstargetprediction.ch) (accessed on 7 March 2024) [33] and STITCH web server (http://stitch.embl.de/) (accessed on 7 March 2024) [34], with “Homo sapiens” as the species of interest to narrow the scope to human-related targets. To encompass the broadest possible interactome and avoid the omission of potential regulatory targets, a probability threshold of ≥0.0 was applied, ensuring an inclusive identification of all predicted interactions for further network analysis. Finally, potential NO inhibition targets were identified by determining the intersection of the datasets comprising “Target related to NO” and “Target of ocotillol,” thereby identifying the key molecular targets associated with anti-inflammatory potential through the NO inhibition pathway.

### 3.5. GO Analysis and KEGG Pathway Analysis

Gene Ontology (GO) analysis and Kyoto Encyclopedia of Genes and Genomes (KEGG) enrichment assessments were conducted using ShinyGO v0.80, the online tool platform http://bioinformatics.sdstate.edu/go/ (accessed on 15 April 2024) with the species set as *Homo sapiens* [35]. The results of Gene Ontology (GO) analysis are categorized into the classifications of biological processes (BP), cellular components (CC), and molecular function (MF). An assessment of the significance of pertinent terminology is conducted utilizing the *p*-value < 0.05 employed for all enrichment evaluations.

### 3.6. Molecular Docking

3D-Structure of specific proteins from identified core targets was collected from the Protein Data Bank (PDB) as indicated in Table 1. Subsequently, the hydrogen atoms were added, followed by the assignment of partial charges. Prior to conducting the molecular docking process, the protocol underwent validation through the re-docking of co-crystal ligands, which were then compared against experimental data. The methodology was considered to be accurate and reliable if the root mean square deviation (RMSD) between the co-crystal and re-docked ligands was below 2 Å [36]. After that, ocotillol was docked into the active site of target proteins using Autodock Vina 1.5.7 [37]. The docking results were then analyzed employing the Discovery Studio Visualizer 24.1.0.23298 software.

### 3.7. Molecular Dynamics

In this study, molecular dynamics simulations were performed utilizing Nanoscale Molecular Dynamics (NAMD) version 2.14 [38]. Initially, the proteins were prepared using Visual Molecular Dynamics (VMD) version 1.9.7, and the ligand structural file was obtained from the CHARMM-GUI server [39]. Subsequently, a cubic water box was constructed and the system was neutralized with Na^+^ and Cl^−^ ions at a concentration of 0.15 M followed by a energy minimization period in 1000 steps, and a simulation period of 100 ns (equivalent to 50 million steps, with a timestep of 2 fs/step) within the NPT ensemble (Pressure: 1 bar and Temperature: 300 K). The results were evaluated based on the root mean square deviation (RMSD) of the protein backbone, the root mean square fluctuation (RMSF) of the alpha carbon (Cα), the radius of gyration (RG) of the Cα, and the solvent accessible surface area (SASA) utilizing VMD 1.9.7.

## 4. Conclusions

This study elucidates the anti-inflammatory potential through in vitro nitric oxide (NO) inhibition of the ocotillol with an IC_50_ value of 20.29 ± 2.1 µg/mL. In-depth in silico studies identified 14 common molecular targets between the compound and genes influencing NO. Molecular docking results revealed that the TACR1 target (ID: 6HLO) exhibited the lowest binding energy. Furthermore, molecular dynamics simulations suggest that ocotillol binds stably within the TACR1 binding pocket. These findings indicate a correlation between in vitro and in silico models, thereby providing a foundation for future in vivo evaluations to confirm the anti-inflammatory mechanism of ocotillol.

## Figures and Tables

**Table 1:** In vitro anti-inflammatory activity and cytotoxicity of ocotillol.

Sample	Concentration (µg/mL)	Cell Viability (%)	NO Inhibition (%)	IC_50_ (µg/mL)
Ocotillol	128	53	77	20.29 ± 2.1
	32	97	70	
	8	100	29	
	2	100	21	
N^G^-Methyl-L-arginine acetate (L-NMMA) *	128	90	92	5.71 ± 0.5
	32	99	85	
	8	99	63	
	2	100	29	

*: positive control.

**Table 2:** Name of protein and ID from PBD.

Name of Protein	Protein Code	ID Protein
MAP kinase p38 alpha	MAPK14	4FA2
Vascular endothelial growth factor receptor 2	KDR	3CJG
TNF-alpha	TNF	2AZ5
Arachidonate 5-lipoxygenase	ALOX5	6N2W
Serine/threonine-protein kinase receptor R3	ACVRL1	4GV1
Neurokinin 2 receptor	TACR2	3HN4
p53-binding protein Mdm-2	MDM2	3JZK
Renin	REN	2V0Z
Insulin-like growth factor I receptor	IGF1R	2ZM3
Neurokinin 1 receptor	TACR1	6HLO
MAP kinase ERK1	MAPK3	4QTB
Peroxisome proliferator-activated receptor gamma	PPARG	2P4Y
Progesterone receptor	PGR	2OVH
Signal transducer and activator of transcription 6	STAT6	9BIG

## Data Availability

The original contributions presented in this study are included in the article. Further inquiries can be directed to the corresponding authors.

## Supplementary Materials

The following supporting information can be downloaded at: https://www.mdpi.com/article/10.3390/ph19030361/s1, Table S1: The genes related to NO biosynthesis were collected from the GeneCards data-base using the searching keyword “Nitric oxide synthesis”; Table S2: OMIM databases; Table S3: SwissTarget; Table S4: Overlapping genes; Table S5: Root Mean Square Deviation (RMSD); Table S6: Root Mean Square Fluctuation (RMSF); Table S7: Solvent Accessible Surface Area (SASA); Table S8: Radius of Gyration.
